# Early dynamic changes in platelet counts and 28-day mortality in sepsis patients: a retrospective cohort study using dynamic latent class model and generalized additive mixture model analysis

**DOI:** 10.3389/fmed.2025.1596134

**Published:** 2025-07-09

**Authors:** Yong Han, Jie Liu, Zhenhua Huang, Haofei Hu, Haiyan Yin

**Affiliations:** ^1^Department of Intensive Care Unit, The First Affiliated Hospital of Jinan University, Jinan University, Guangzhou, China; ^2^Department of Emergency, Shenzhen Second People's Hospital, The First Affiliated Hospital of Shenzhen University, Shenzhen, Guangdong, China; ^3^Department of Nephrology, Shenzhen Second People's Hospital, The First Affiliated Hospital of Shenzhen University, Shenzhen, Guangdong, China

**Keywords:** sepsis, dynamic changes in platelet counts, mortality, dynamic latent class model, generalized additive mixture model

## Abstract

**Objective:**

This study investigates the relationship between early dynamic changes in platelet (PLT) counts and 28-day mortality in Chinese patients with sepsis, addressing the limitations of previous studies that focused on single baseline measurements.

**Methods:**

In this retrospective cohort study, 266 sepsis patients admitted to Shenzhen Second People’s Hospital from January 2023 to December 2024 were included. A dynamic latent class model analyzed the patterns of PLT count changes during the first week of hospitalization. The Cox proportional hazards regression model assessed the link between these dynamic changes and 28-day mortality, supported by sensitivity and subgroup analyses for robustness. The GAMM model compared PLT change trajectories over 7 days between the mortality and survival groups.

**Results:**

After adjusting for various variables, participants with gradually increasing PLT counts (class 2), decreasing counts (class 3), and persistently low counts (class 4) had hazard ratios (HRs) for 28-day mortality of 1.687 (95% CI:0.380, 7.494), 3.710 (95% CI:1.124, 12.251), and 4.258 (95% CI:1.435, 12.636) respectively, compared to those with persistently high PLT counts (class 1). The GAMM model revealed that PLT counts for patients who died were significantly lower and had a downward trend, while the survival group’s counts trended upward; the difference between the two groups generally exhibited an upward trend after admission, with a calculated average daily increase of 12.919 × 10^9^/L.

**Conclusion:**

Early dynamic changes in PLT counts (1–7 days) are independently associated with 28-day mortality in sepsis patients. Those with low and declining PLT counts are at a higher risk. By dynamically monitoring early changes in PLT may help identify high-risk patients and inform personalized treatment strategies, improving outcomes.

## Introduction

Sepsis is a severe syndrome resulting from a misregulated response of the host to infection, impacting millions of individuals across the globe each year (1, 2). Despite notable advancements in critical care practices, sepsis continues to be a leading cause of mortality worldwide, placing a significant strain on both society and healthcare systems (3, 4). Recent epidemiological studies reveal that the mortality rate associated with sepsis in intensive care units varies between 20 and 40%, influenced by geographic location and patient demographics (5, 6). Additionally, those who survive sepsis frequently suffer from long-term complications, including both physical and cognitive impairments, along with an elevated risk of subsequent death and hospital readmission (7–9). These findings highlight the pressing need for dependable prognostic markers that can aid in the early identification and management of patients with a high risk of sepsis.

Platelets (PLT) are crucial for coagulation, inflammation, and immune responses, all of which are closely linked to the prognosis of sepsis patients (10–13). Inflammation and endothelial activation due to sepsis can trigger PLT activation and consumption, leading to PLT sequestration and destruction (14). Additionally, sepsis impairs megakaryocyte functionality, which reduces PLT production and further aggravates thrombocytopenia (15). Beyond their role in hemostasis, PLT is also a key player in immune regulation, and PLT depletion may impair the host’s defense against pathogens and exacerbate the inflammatory response (16). Numerous studies have demonstrated significant associations between PLT counts and sepsis severity or mortality (17–19). However, some studies have failed to establish this relationship, and even contradictory findings have been reported (20, 21). One possible explanation is that previous studies often relied on single baseline PLT measurements, which may not reflect the dynamic and heterogeneous clinical course of sepsis.

There is substantial evidence that PLT counts fluctuate over time, typically reaching their nadir on the third to fifth day of hospitalization in sepsis patients. This suggests that, in addition to baseline PLT counts, the dynamic trajectory of PLT changes over time may play a crucial role in determining patient outcomes. Unfortunately, previous research has primarily focused on the relationship between single-time-point PLT measurements and sepsis outcomes, with limited attention given to the prognostic value of longitudinal PLT trajectories derived from repeated measurements in routine clinical practice. To date, only two studies have analyzed the association between PLT trajectories during the first 4 days of intensive care unit (ICU) admission and in-hospital mortality using publicly available datasets (22, 23). Therefore, this study aims to conduct a retrospective cohort analysis to investigate the impact of baseline PLT counts and their dynamic trajectories during the first week on 28-day mortality in sepsis patients presenting to the emergency department.

## Methods

### Study design and study population

This retrospective cohort study systematically included sepsis patients who were admitted to the emergency department from January 2023 to December 2024. The main independent variables examined were PLT assessed upon admission and the dynamic changes in PLT observed during the first week of hospitalization. The primary outcome of interest was the 28-day mortality among sepsis patients.

**Figure 1:**
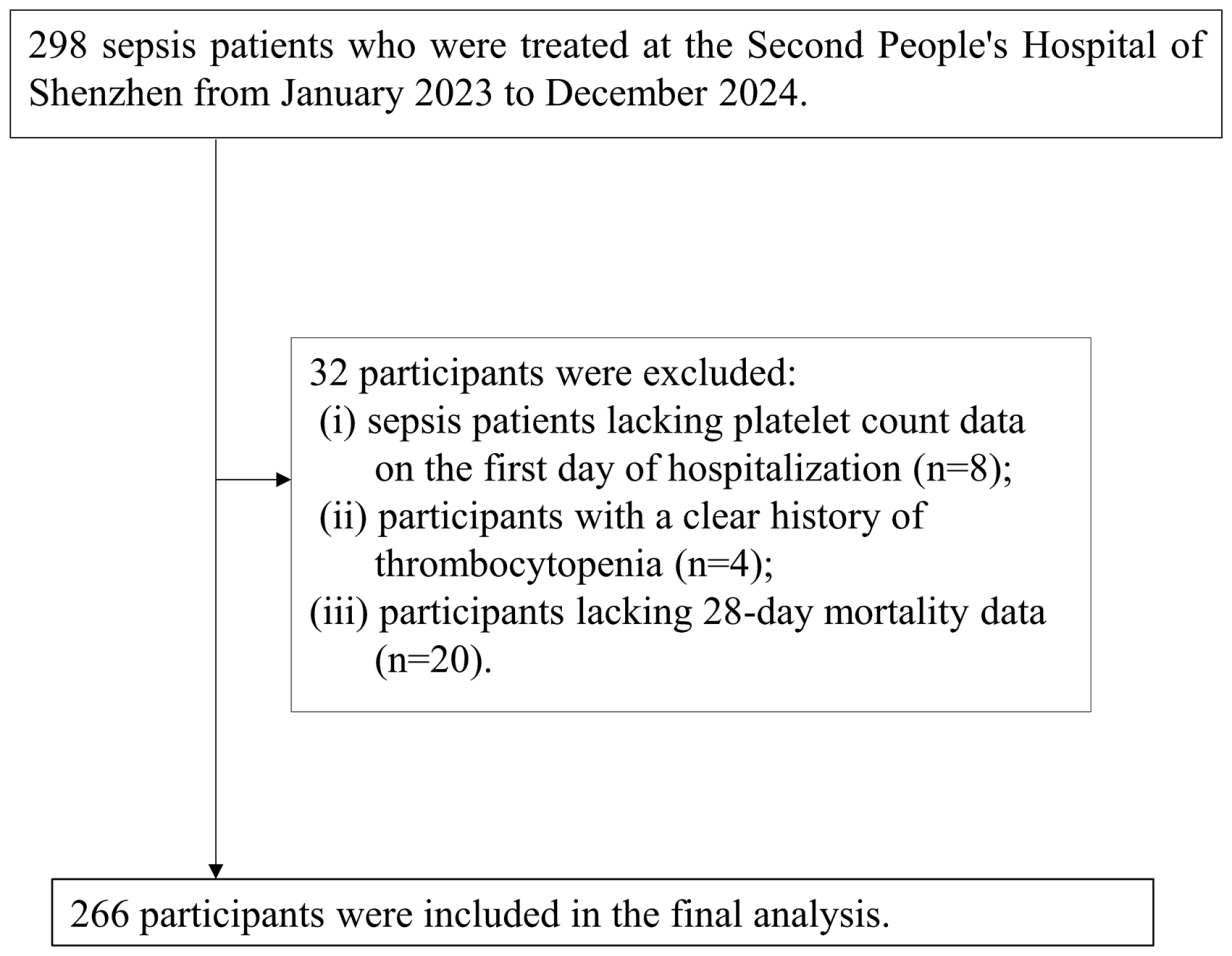
Flowchart of the selection process for study participants.

This study focused on sepsis patients who were enrolled consecutively from January 2023 to December 2024 at Shenzhen Second People’s Hospital, with diagnoses made according to established guidelines. Initially, a cohort of 298 sepsis patients was identified. The inclusion criteria were: (a) patients aged 18 years or older and (b) those meeting the diagnostic standards for sepsis as outlined in the Third International Consensus Definitions for Sepsis and Septic Shock (Sepsis-3) (1). Following this, 32 participants were excluded based on specific criteria: (i) 8 patients without PLT count data on the first day of hospitalization; (ii) 4 patients with a documented history of thrombocytopenia; and (iii) 20 patients lacking data on 28-day mortality. Ultimately, 266 participants were retained for the final analysis. The process of participant selection is illustrated in Figure 1.

### Ethical approval and consent

Approval for this study was granted by the Clinical Research Ethics Committee at Shenzhen Second People’s Hospital (Ethics Approval Number: 2023-93325-01PJ). Given the retrospective nature of the research, the Ethics Committee waived the requirement for informed consent. Furthermore, this study was conducted in strict accordance with the principles outlined in the Declaration of Helsinki and complied with the relevant ethical standards and regulations specified in the declaration.

### Dynamic patterns of PLT changes within one week in sepsis patients

The independent variables in this study include PLT counts at admission and their dynamic trajectories within the first week of hospitalization. It is important to note that PLT levels were not measured daily after admission, but the number of repeated measurements was at least two or more. This study classified the dynamic patterns of PLT changes within the first week of hospitalization in sepsis patients using the Dynamic Latent Class Model (DLCM). This type of Model is an extension of traditional Latent Class Analysis, incorporating time dynamics (dynamic changes) and latent class analysis to handle observational data collected at multiple time points. Unlike static latent class models, the core feature of dynamic latent class models lies in their ability to capture the transition patterns of classified groups (latent classes) and the evolving characteristics of these groups over time (24, 25).

### 28-day mortality assessment

The primary endpoint of this study was 28-day mortality. Data on all-cause mortality were collected primarily through the electronic medical record system and post-discharge telephone follow-ups. The post-discharge follow-up information was obtained by trained follow-up personnel through interviews or telephone conversations.

### Covariates

The selection of covariates was based on previous studies and our clinical expertise (26–28). Continuous variables: white blood cell count (WBC), cardiac troponin I (cTnI), age, total bilirubin (TBIL), red blood cell distribution width (RDW), procalcitonin (PCT), albumin (ALB), lymphocyte count (LYM), aspartate aminotransferase (AST), lactate (Lac), hemoglobin (HGB), neutrophil count (NEU), D-dimer (DD), serum creatinine (Scr), blood urea nitrogen (BUN), mean arterial pressure (MAP), fibrinogen (FIB), alanine aminotransferase (ALT), carbon dioxide combining power (CO_2_CP), Sequential Organ Failure Assessment score (SOFA score); C-reactive protein (CRP), base excess (BE), 24-h intake (24h_in), 24-h output (24h_out), 24-h fluid balance, and Acute Physiology and Chronic Health Evaluation II (APACHE) score. Categorical variables: coronary heart disease (CHD), mechanical ventilation (MV), Infection site, diabetes mellitus (DM), sex, chronic kidney disease (CKD), use of antibiotics within 1 h (ABX < 1 h), history of stroke, hypertension (HTN), congestive heart failure (CHF), shock, chronic obstructive pulmonary disease (COPD), malignancy, use of vasoactive drugs (VAD), and Continuous Renal Replacement Therapy (CRRT).

### Data collection and measurement

At admission, trained research coordinators collected baseline data on demographics and medical history, including information on previous stroke, DM, hypertension, atrial fibrillation, and heart disease. The severity of illness in sepsis patients was assessed using the APACHE II score and SOFA score by trained Emergency ICU physicians upon admission. Fluid intake and output were recorded by experienced Emergency ICU nurses. Baseline blood samples were collected within hours of admission and analyzed in the clinical laboratory of Shenzhen Second People’s Hospital. Analytical instruments included the Beckman 5,800 automated analyzer and the Mindray 5,180 hematology analyzer. Experienced technicians performed rigorous quality control and measurement of laboratory parameters while maintaining patient confidentiality regarding baseline information. Repeat measurement data were obtained from re-examinations conducted during the 24-h hospitalization period following admission.

### Handling of missing data

In our study, missing baseline data were observed for the following data points: SOFA score (23, 8.65%), Infection site (22, 8.27%), CHF (2, 0.75%), CKD (2, 0.75%), WBC (20, 7.52%), HGB (20, 7.52%), RDW (20, 7.52%), LYM (22, 8.27%), NEU (22, 8.27%), Lac (35, 13.16%), PCT (37, 13.91%), CRP (37, 13.91%), FBG (38, 14.29%), MAP (15, 5.64%), Scr (37, 13.91%), FPG (27, 10.15%), ALB (40, 15.04%), HDL-c (40, 15.04%), LDL-c (40, 15.04%), TG (40, 15.04%), and (40, 15.04%). Missing data in the modeling process can compromise the statistical validity of the target sample. To minimize bias introduced by missing variables, multiple imputations were performed to address the missing data (29, 30). Covariates in the estimation model included stroke, WBC, NEU, LYM, HGB, RDW, ALB, TBIL, AST, ALT, BUN, Scr, CO_2_CP, Lac, cTnI, BNP, FIB, DD, PCT, CRP, BE, 24h_in, infection site,24h_out, 24-h fluid balance, MAP, APACHE II score, SOFA score, CHD, DM, ABX < 1 h, HTN, CHF, CRI, COPD, VAD, MV, and CRRT (using linear regression with 10 iterations). The analysis of missing data was conducted under the assumption of missing at random (MAR) (30).

### Statistical analysis

Statistical analyses were conducted using R software version 3.4.3 and Empower(R) version 4.2. Two-tailed tests were employed, with significance defined as a *p*-value of less than 0.05. The DLCM was utilized to categorize the changes in PLT based on repeated measurements of PLT counts collected from the day of admission through day 7 of hospitalization for patients with sepsis. Continuous variables were reported as medians with interquartile ranges or mean with standard deviations (SD). Categorical data were presented as frequencies and percentages. The χ^2^ test was applied to assess differences in categorical variables across the classifications of PLT trajectories, while Analysis of Variance (ANOVA) and the Kruskal-Wallis H test were implemented to compare continuous variables among the groups.

Three Cox proportional hazards regression models were developed to assess the relationship between baseline PLT counts (measured at admission) and 28-day mortality in sepsis patients, calculating hazard ratios (HRs) and 95% confidence intervals (CIs). The models were structured as follows: Model I was unadjusted; Model II included adjustments for age and sex; while Model III additionally adjusted for age, sex, stroke, WBC, ALB, TBIL, Scr, CO₂CP, Lac, cTnI, FIB, CRP, MAP, ABX < 1 h, 24-h fluid balance, SOFA score, infection site, CKD, DM, CHF, COPD, VAD, MV, and CRRT. After categorizing the changes in PLT counts, Cox proportional hazards regression models were employed to explore the association between these classifications and the risk of 28-day mortality. Again, Model I remained unadjusted, Model II accounted for age and sex, and Model III involved the same adjustments as before. In addition, the Generalized Additive Mixed Model (GAMM) was utilized to investigate the relationship between dynamic PLT counts during the first week and 28-day mortality, examining differences in PLT counts between survivors and non-survivors throughout this period. GAMM is commonly used for analyzing repeatedly collected data, particularly when data sets contain missing values or irregular collection patterns (31, 32).

To ensure the robustness of the study findings, a comprehensive series of sensitivity analyses was conducted. Previous studies have shown a significant association between DM, CKD, and the prognosis of sepsis (33, 34). In this context, the relationship between patterns of dynamic changes in PLT and 28-day mortality was further explored, specifically in sepsis patients without a history of CKD, or DM. Additionally, to assess the validity of multiple imputation data, a validation analysis was conducted based on the original data before imputation. Meanwhile, E-values were also calculated to evaluate the impact of unknown or unmeasured factors on the relationship between early dynamic PLT changes in sepsis and 28-day mortality (35).

A stratified Cox proportional hazards regression model was employed for subgroup analysis based on factors such as age, sex, CHD, CKD, DM, hypertension, and shock. Age was initially dichotomized at clinical thresholds: <60 years and ≥60 years. In addition to these stratification variables, the model was adjusted for other covariates, including age, sex, stroke, WBC, ALB, TBIL, Scr, CO₂CP, Lac, cTnI, FIB, CRP, MAP, ABX < 1 h, 24-h fluid balance, SOFA score, infection site, CKD, DM, CHF, COPD, VAD, MV, and CRRT. To evaluate potential interactions, likelihood ratio tests compared models with and without interaction terms.

## Results

### Classification of one-week dynamic PLT changes in sepsis patients based on dynamic latent class model

**Figure 2:**
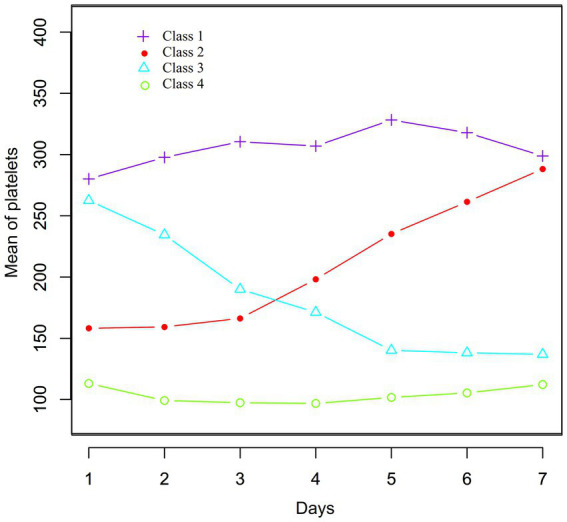
Data visualization of the pattern of dynamic changes in PLT counts in the early stage (1–7 days) in sepsis patients.

Through dynamic latent class analysis, PLT changes in sepsis patients during the first 7 days of hospitalization were classified into four categories: Class 1 (*n* = 70): Mean PLT values from day 1 to day 7 after admission were 280.09, 297.72, 310.50, 306.96, 328.26, 317.89, and 298.32, showing consistently high PLT levels. Class 2 (*n* = 33): PLT values from day 1 to day 7 after admission were 158.16, 159.27, 166.27, 198.19, 235.20, 261.36, and 283.91, indicating a gradually increasing trend from a relatively low baseline level. Class 3 (*n* = 44): PLT values from day 1 to day 7 after admission were 262.52, 234.46, 190.03, 171.21, 140.20, 138.12, and 131.44, showing a gradual decreasing trend from a relatively high baseline level. Class 4 (*n* = 119): PLT values from day 1 to day 7 after admission were 113.13, 99.17, 97.43, 96.82, 101.74, 105.41, and 111.17, indicating consistently low PLT levels (Figure 2).

### Participant characteristics

A total of 266 participants were included in the analysis, comprising 161 males and 105 females, with a mean age (± SD) of 67.86 ± 19.00 years. Table 1 summarized the baseline characteristics of participants classified according to PLT dynamic changes. Compared to Class 1 (persistently low PLT count), participants in other classes had higher levels of age, RDW, BUN, Scr, BNP, DD, SOFA score, Lac, 24-h intake, and APACHE II scores, while ALB, FBG, 24-h output, and MAP levels were lower. Additionally, participants in other classes had a higher proportion of hypertension, urinary tract infection, males, CKD, history of stroke, shock, VAD, and MV compared to Class 1 (Table 1).

**Table 1:** The baseline characteristics of participants.

Classification of dynamic changes in PLT within 7 days					
Characteristic	Class 1	Class 2	Class 3	Class 4	*p*-value
*N*	70	33	44	119	
Age (years)	63.27 ± 17.95	63.56 ± 20.52	67.59 ± 18.17	71.76 ± 18.00	0.014
WBC (×10^9^/L)	13.45 ± 7.25	13.87 ± 5.99	15.70 ± 7.67	13.02 ± 12.10	0.473
PLT (×10^9^/L baseline)	268.27 ± 47.10	268.27 ± 47.10	250.70 ± 57.89	100.96 ± 53.52	<0.001
NEU (×10^9^/L)	9.94 (6.50–14.12)	10.38 (7.40–14.84)	13.03 (6.90–18.93)	9.43 (5.55–12.55)	0.288
HGB (g/L)	110.02 ± 19.31	102.82 ± 28.74	108.07 ± 24.85	101.49 ± 23.66	0.079
Lym (×10^9^/L)	1.05 (0.68–1.28)	0.94 (0.81–1.27)	1.00 (0.61–1.52)	0.77 (0.48–1.23)	0.341
RDW (%)	14.27 ± 2.75	14.88 ± 3.56	15.22 ± 3.62	15.75 ± 4.30	0.002
ALB (g/L)	30.35 ± 6.72	32.23 ± 4.95	29.85 ± 6.33	28.27 ± 6.53	0.009
TBIL (U/L)	16.01 (10.22–27.45)	11.30 (8.10–22.60)	14.00 (13.40–15.75)	15.70 (10.30–24.73)	0.100
AST (U/L)	30.00 (17.00–115.00)	36.00 (21.00–102.52)	31.50 (18.00–76.06)	39.00 (25.00–72.50)	0.506
ALT (U/L)	31.00 (18.50–64.30)	23.30 (8.00–36.00)	18.03 (13.28–40.25)	34.00 (18.50–58.00)	0.085
BUN (mmol/L)	5.60 (3.80–11.52)	5.80 (3.83–8.60)	9.60 (4.70–20.44)	13.20 (7.50–23.07)	<0.001
Scr (umol/L)	74.30 (65.20–127.25)	84.77 (63.10–129.05)	98.30 (73.22–170.72)	130.90 (85.30–201.35)	0.005
CO₂CP (mmol/L)	21.68 ± 4.93	22.67 ± 5.04	21.52 ± 4.57	21.91 ± 5.83	0.794
CTnI (μg/L)	0.01 (0.01–0.08)	0.01 (0.01–0.01)	0.02 (0.01–0.05)	0.02 (0.01–0.10)	0.287
BNP (pg/mL)	471.00 (223.00–1170.00)	2165.00 (373.00–4752.50)	1595.00 (700.75–4940.00)	3980.00 (1870.00–11577.56)	<0.001
DD (mg/L)	2.13 (1.51–3.50)	2.75 (0.93–6.52)	2.77 (0.84–5.41)	7.23 (2.74–14.50)	<0.001
FIB (g/L)	5.64 ± 2.13	6.57 ± 2.39	5.30 ± 1.83	4.16 ± 2.04	<0.001
PCT (ng/mL)	22.12 ± 40.12	12.89 ± 29.90	15.32 ± 30.01	26.70 ± 90.69	0.644
CRP (mg/L)	146.39 ± 96.63	129.23 ± 87.64	110.25 ± 93.36	115.45 ± 93.78	0.113
BE (mmol/L)	−2.83 ± 3.92	−1.35 ± 3.65	−3.87 ± 6.00	−3.55 ± 5.40	0.177
Lac (mmol/L)	1.70 ± 1.05	1.89 ± 1.06	2.79 ± 2.71	2.70 ± 2.56	0.005
FIO2	84.98 ± 99.57	112.56 ± 148.10	164.07 ± 159.85	188.00 ± 202.84	<0.001
24h_in (ml)	471.00 (223.00–1170.00)	471.00 (223.00–1170.00)	1595.00 (700.75–4940.00)	3980.00 (1870.00–11577.56)	0.064
24h_out t (ml)	1130.25 (350.00–1950.50)	1040.50 (150.00–1861.50)	997.3 (732.38–1423.62)	1351.00 (439.50–1556.00)	0.049
24-h fluid balance (ml)	−592.15 (−414.00–725.15)	−728.90 (−576.90–892.90)	1876.90 (168.75–3786.38)	2780.40 (1562.00–5201.90)	0.010
MAP (mmHg)	78.10 ± 13.91	77.79 ± 15.38	71.55 ± 20.29	67.76 ± 14.94	0.698
APACHE II score	16.48 (10.67–21.24)	20.20 (13.42–28.63)	21.75 (16.25–29.00)	25.00 (16.00–31.50)	<0.001
SOFA score	10.82 ± 2.83	11.89 ± 2.65	12.09 ± 2.07	13.83 ± 2.58	<0.001
Sex					0.008
Male	31 (44.29%)	19 (57.58%)	31 (70.45%)	80 (67.23%)	
Female	39 (55.71%)	14 (42.42%)	13 (29.55%)	39 (32.77%)	
Hypertension (*n*, %)	27 (38.57%)	13 (39.39%)	19 (43.18%)	69 (57.98%)	0.035
CHD (*n*, %)	8 (11.43%)	2 (6.06%)	7 (15.91%)	21 (17.65%)	0.323
CHF (*n*, %)	5 (7.14%)	3 (9.09%)	8 (18.18%)	19 (15.97%)	0.213
CKD (*n*, %)	3 (4.28%)	4 (12.12%)	8 (18.18%)	24 (20.16%)	0.009
DM (*n*, %)	17 (24.29%)	9 (27.27%)	14 (31.82%)	42 (35.29%)	0.434
Stroke (*n*, %)	4 (5.71%)	7 (21.21%)	10 (22.73%)	25 (21.01%)	0.030
COPD (*n*, %)	1 (1.43%)	1 (3.03%)	1 (2.27%)	11 (9.24%)	0.073
Malignancy (*n*, %)	1 (1.43%)	3 (9.09%)	5 (11.36%)	25 (21.01%)	0.001
Shock (*n*, %)	18 (25.71%)	11 (33.33%)	28 (63.64%)	72 (60.50%)	<0.001
VAD (*n*, %)	13 (18.57%)	9 (27.27%)	28 (63.64%)	66 (55.46%)	<0.001
MV (*n*, %)	5 (7.14%)	8 (24.24%)	27 (61.36%)	61 (51.26%)	<0.001
ABX<1 h (*n*, %)	41 (58.57%)	22 (66.7%)	32 (72.73%)	88 (73.95%)	0.105
CRRT (*n*, %)	2 (2.86%)	5 (15.15%)	12 (27.27%)	33 (27.73%)	<0.001
Infection site					<0.001
Pulmonary	34 (48.57%)	5 (15.15%)	11 (25.00%)	18 (15.13%)	
Abdominal cavity	12 (17.14%)	3 (9.09%)	2 (4.55%)	12 (10.08%)	
Urinary	17 (24.29%)	25 (75.76%)	27 (61.36%)	84 (70.59%)	
Others	7 (10.00%)	0 (0.00%)	4 (9.09%)	5 (4.20%)	

Continuous variables were summarized as mean (SD) or medians (quartile interval); categorical variables were displayed as percentages (%). *N*, number; WBC, white blood cell count; PLT, platelet count; NEU, neutrophil count; Lym, lymphocyte count; HGB, hemoglobin; RDW, red blood cell distribution width; ALB, albumin; TBIL, total bilirubin; AST, aspartate aminotransferase; ALT, alanine aminotransferase; BUN, blood urea nitrogen; Scr, serum creatinine; CO₂CP, carbon-dioxide combining power; Lac, lactate; CTnI, cardiac troponin I; BNP, brain natriuretic peptide; FIB, fibrinogen; PCT, procalcitonin; CRP, C-reactive protein; BE, base excess; FIO₂, fraction of inspired oxygen; 24h_in, 24-h intake; 24h_out, 24-h output; 24-h fluid balance; MAP, mean arterial pressure; APACHE II, Acute Physiology and Chronic Health Evaluation II score; SOFA score, Sequential Organ Failure Assessment score; VAD, vasoactive drugs; MV, mechanical ventilation; ABX<1 h, antibiotics initiated within 1 h; CRRT, continuous renal replacement therapy; CHD, coronary heart disease; CHF, congestive heart failure; CKD, chronic kidney disease; DM, diabetes mellitus; COPD, chronic obstructive pulmonary disease.

### 28-day mortality in sepsis patients

During a mean follow-up duration of 24.38 days, 67 participants succumbed, resulting in an overall mortality of 25.19%. The mortality for each classification was 5.17% for Class 1, 12.12% for Class 2, 27.27% for Class 3, and 39.50% for Class 4. In comparison to sepsis patients in Class 1, the 28-day mortality was markedly elevated in the other classes (Table 2).

**Table 2:** 28-day mortality for participants with sepsis.

Classification of dynamic changes in PLT	Participants (*n*)	Death events (*n*)	Mortality (95% CI) (%)
Total	266	67	25.19 (19.94–30.44)
Class 1	70	4	5.71 (0.14–11.29)
Class2	33	4	12.12 (0.37–23.87)
Class3	44	12	27.27 (13.58–40.97)
Class4	119	47	39.50 (30.58–48.41)
P for trend			<0.001

CI, confidence.

**Figure 3:**
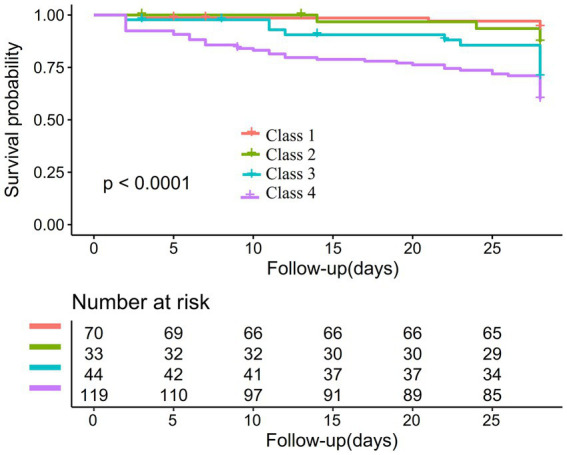
Kaplan–Meier survival curves stratified by classifications of PLT dynamic changes.

In addition, the Kaplan–Meier survival curves illustrate survival probabilities according to classifications of PLT dynamic changes. Importantly, participants in classes 3 and 4 exhibited significantly decreased survival probabilities when compared to those in classes 1 and 2, with class 4 participants demonstrating the greatest risk of mortality (Figure 3).

### Relationship between baseline PLT count and 28-day mortality in sepsis patients

To explore the association between baseline PLT counts at admission and 28-day mortality, three Cox proportional hazards regression models were developed, as outlined in Table 3. In Model I, a 10 × 10^9^/L increase in PLT was linked to a 4% decrease in mortality risk among sepsis patients (HR = 0.960, 95% CI: 0.930–0.991). Model II provided similar findings, indicating that each 10 × 10^9^/L rise in PLT correlated with a 3.9% reduction in mortality risk (HR = 0.961, 95% CI: 0.931–0.991). Furthermore, in the fully adjusted Model III, each 10 × 10^9^/L increase in PLT was associated with a 4.3% decline in mortality risk (HR = 0.957, 95% CI: 0.928, 0.988).

**Table 3:** Relationship between admission PLT count and 28-day mortality in sepsis patients in different models.

Exposure	Model I (HR, 95%CI) *p*	Model II (HR, 95%CI) *p*	Model III (HR, 95%CI) *p*
PLT (per 10 × 10^9^/L)	0.960 (0.930, 0.991) 0.011	0.961 (0.931, 0.991) 0.013	0.957 (0.928, 0.988) 0.006
PLT group			
<100*10^9^/L	Ref	Ref	Ref
100–200*10^9^/L	0.673 (0.388, 1.167) 0.158	0.715 (0.408, 1.251) 0.240	0.817 (0.456, 1.462) 0.496
≥200*10^9^/L	0.448 (0.228, 0.880) 0.020	0.479 (0.243, 0.946) 0.034	0.499 (0.250, 0.996) 0.048
P for trend	<0.001	<0.001	<0.001

Model I did not adjust for any covariates.

Model II adjusted for age and sex.

Model III adjusted for age, sex, stroke, WBC, ALB, TBIL, Scr, CO₂CP, Lac, CTnI, FIB, CRP, MAP, ABX<1 h, 24-h fluid balance, SOFA score, infection site, CKD, DM, CHF, COPD, VAD, MV, and CRRT.

HR, hazard ratio; Ref, reference; CI, confidence.

Additionally, participants were categorized into three groups according to their baseline PLT counts: <100, 100–200, and ≥200. In the multivariable-adjusted model, using those with PLT < 100 as the reference category, the HRs for 28-day mortality were 0.817 (95% CI: 0.456, 1.462) for the 100–200 group and 0.499 (95% CI: 0.250, 0.996) for the ≥200 group. These findings suggest that compared to sepsis patients with PLT < 100, those in the 100–200 range had a non-significantly 18.3% lower mortality risk. In addition, patients with PLT ≥ 200 exhibited a 50.1% reduction in mortality risk. This trend aligns with the results from the analysis treating baseline PLT as a continuous variable (P for trend<0.001).

### The relationship between the patterns of dynamic changes in PLT within the first week of admission in sepsis patients and 28-day mortality

Table 4 presented the relationship between patterns of PLT dynamic changes and 28-day mortality in patients with sepsis. In Model I, when compared to class 1, the HRs (95% CI) for 28-day mortality were 2.170 (0.543, 8.676) for class 2, 5.286 (1.704, 16.391) for class 3, and 8.367 (3.013, 23.230) for class 4. In Model II, the HRs compared to class 1 were 2.065 (95% CI: 0.515, 8.271) for class 2, 4.778 (95% CI: 1.533, 14.897) for class 3, and 7.435 (95% CI: 2.648, 20.880) for class 4. Model III, which accounted for more potential confounders, revealed adjusted HRs of 1.687 (95% CI:0.380, 7.494) for class 2, 3.710 (95% CI:1.124, 12.251) for class 3, and 4.258 (95% CI:1.435, 12.636) for class 4, in comparison to class 1. These findings indicate that relative to class 1 (patients with consistently high PLT levels), the 28-day mortality risk for class 2 (those with a low baseline PLT count followed by a gradual increase) was non-significantly increased (68.7%). Relative to class 1, class 3 patients (those with high baseline PLT levels followed by a gradual decline) had a 2.710-fold increased mortality risk, while class 4 patients (those with persistently low PLT levels) had a 3.258-fold increased mortality risk.

**Table 4:** The relationship between dynamic change of PLT and 28-day mortality in different models.

Exposure	Model I (HR, 95%CI) *p*	Model II (HR, 95%CI) *p*	Model III (HR, 95%CI) *p*
Classification of dynamic changes in PLT within 7 days			
Class 1	Ref	Ref	Ref
Class 2	2.170 (0.543, 8.676) 0.273	2.065 (0.515, 8.271) 0.306	1.687 (0.380, 7.494) 0.492
Class 3	5.286 (1.704, 16.391) 0.004	4.778 (1.533, 14.897) 0.007	3.710 (1.124, 12.251) 0.031
Class 4	8.367 (3.013, 23.230) < 0.001	7.435 (2.648, 20.880) < 0.001	4.258 (1.435, 12.636) 0.009

Model I did not adjust for any covariates.

Model II adjusted for age and sex.

Model III adjusted for age, sex, stroke, WBC, ALB, TBIL, Scr, CO₂CP, Lac, CTnI, FIB, CRP, MAP, ABX<1 h, 24-h fluid balance, SOFA score, infection site, CKD, DM, CHF, COPD, VAD, MV, and CRRT.

HR, hazard ratio; Ref, reference; CI, confidence.

### Relationship between early (1–7 days) changes in PLT count and 28-day mortality in patients with sepsis as derived by generalized additive mixture modeling

**Figure 4:**
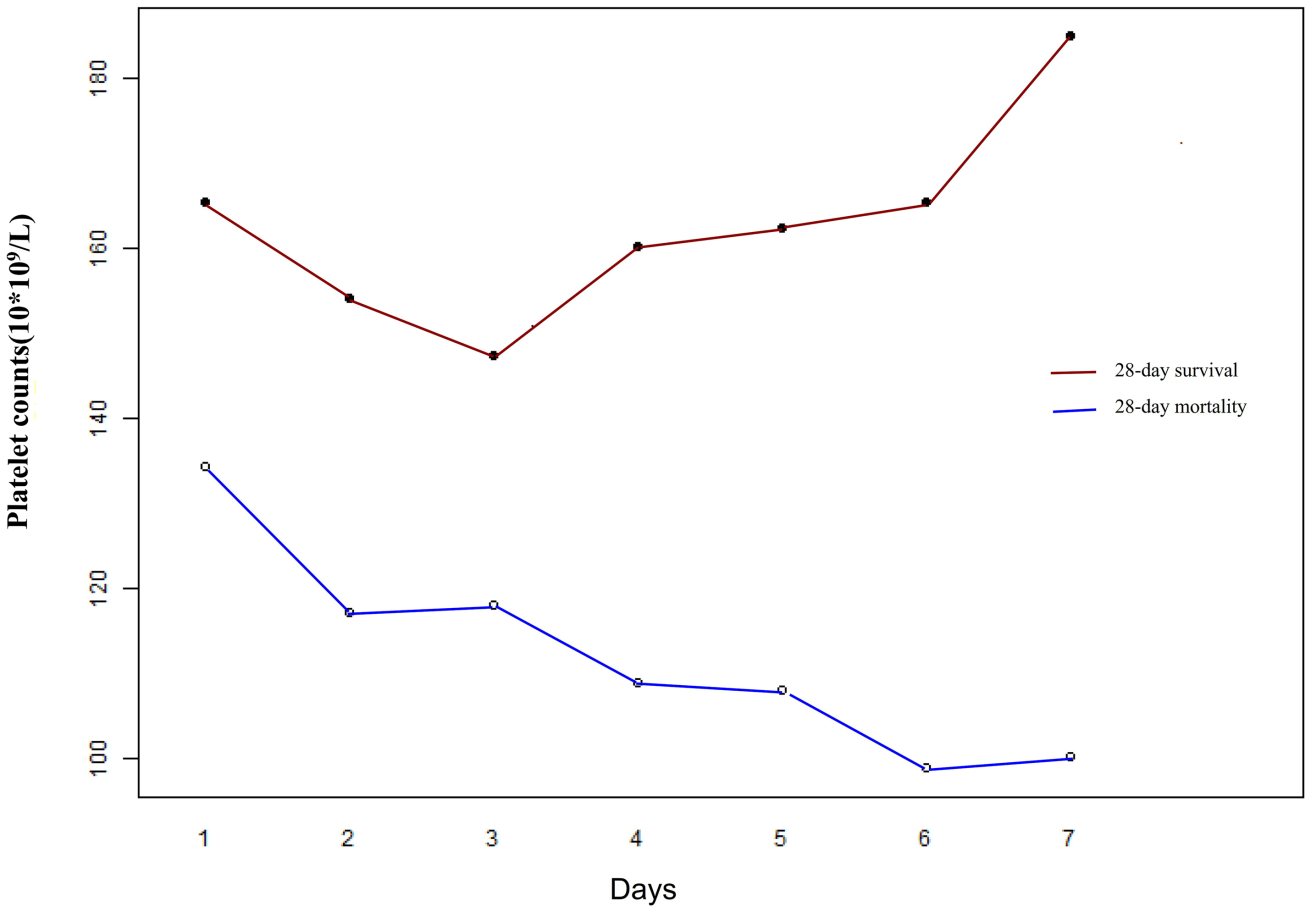
Early dynamic changes in PLT counts of septic patients in the 28-day survival and 28-day mortality groups.

The GAMM model was used to illustrate the trajectories of PLT counts over time for patients in the 28-day survival and mortality groups, adjusting for confounding factors such as age, sex, stroke, WBC, Lac, cTnI, FIB, ABX < 1 h, 24-h fluid balance, SOFA score, infection site, CRP, MAP, CHD, DM, CHF, MV, and CRRT (Figure 4). The baseline PLT count in the survival group was relatively high, demonstrating an upward trend over time, while the mortality group had a lower baseline PLT count, showing a downward trend over time. Table 5 showed the relationship between the early changes in PLT counts (1–7 days) of septic patients and 28-day mortality. The analysis revealed that PLT counts in patients who died within 28 days were significantly lower than those who survived and displayed a downward trend. The difference between the two groups exhibited an increasing trend after admission, with an average daily rise of 12.947 × 10^9^/L. After adjusting for various variables, this increase was determined to be 12.919 × 10^9^/L, indicating robust findings.

**Table 5:** Relationship between early (1–7 days) changes in PLT count (10^9^/L) and 28-day mortality in patients with sepsis as derived by generalized additive mixture modeling (GAMM).

Outcome: PLT	Model I		Model II	
	*β* (95%CI)	*p*-value	*β* (95%CI)	*p*-value
Intercept	151.063 (139.823, 162.303)	< 0.001	178.709 (135.177, 222.241)	< 0.001
Day	4.931 (3.602, 6.259)	< 0.001	4.930 (3.600, 6.260)	< 0.001
Mortality	−12.441 (−34.961, 10.078)	0.280	−10.679 (−33.641, 12.284)	0.363
Day × Mortality	−12.947 (−15.592, −10.301)	< 0.001	−12.919 (−15.567, −10.270)	< 0.001

Intercept, the mean of PLT count at day = 1 and mortality = 0; Day, the mean of the increasing of PLT count at mortality = 1 over time (daily); Mortality, the difference of PLT count at day = 1 between the group of mortality = 1 and the group of mortality = 0; Day × mortality, the average increasing in PLT count daily under the condition of the group of mortality = 1 compared with the group of mortality = 0. Model I: Model I did not adjust for any covariates. Model II adjusted for age, sex, stroke, WBC, Lac, CTnI, FIB, ABX<1 h, 24-h fluid balance, SOFA score, infection site, CRP, MAP, CHD, DM, CHF, MV, and CRRT; CI, confidence interval.

### Sensitivity analysis

Several sensitivity analyses were performed to assess the robustness of the association between dynamic changes in PLT counts and 28-day mortality, as detailed in Table 6. First, an analysis was conducted on sepsis patients without CKD, controlling for various factors such as age, sex, stroke, WBC, ALB, TBIL, Scr, CO₂CP, Lac, cTnI, FIB, CRP, MAP, ABX < 1 h, 24-h fluid balance, SOFA score, infection site, CKD, DM, CHF, COPD, VAD, MV, and CRRT. The findings indicated that, with class 1 as the baseline, the adjusted HRs (95% CI) were 1.536 (0.342, 6.890) for class 2, 3.341 (1.054, 10.592) for class 3 and 5.022 (1.752, 14.396) for class 4. Second, when patients with DM were excluded, similar results were observed after adjusting for confounding variables. Using class 1 as the reference, the adjusted HRs (95% CI) were 1.672 (0.305, 9.170) for class 2, 2.840 (1.696, 9.593) for class 3, and 3.362 (1.330, 9.159) for class 4. Overall, the sensitivity analyses corroborated the findings from the entire population, suggesting that the main conclusions of this study are dependable.

**Table 6:** Relationship between classes of dynamic changes in PLT and 28-day mortality risk in different sensitivity analyses.

Exposure	Model I (HR, 95%CI) *p*	Model II (HR, 95%CI) *p*
Classification of dynamic changes in PLT within 7 days		
Class 1	Ref	Ref
Class 2	1.536 (0.342, 6.890) 0.575	1.672 (0.305, 9.170) 0.554
Class 3	3.341 (1.054, 10.592) 0.040	2.840 (1.696, 9.593) 0.046
Class 4	5.022 (1.752, 14.396) 0.002	3.362 (1.330, 9.159) 0.006

Model I was a sensitivity analysis in participants without CKD, adjusted for included age, sex, stroke, WBC, ALB, TBIL, Scr, CO₂CP, Lac, CTnI, FIB, CRP, ABX<1 h, 24-h fluid balance, SOFA score, infection site, MAP, CHF, COPD, VAD, MV, and CRRT.

Model II was a sensitivity analysis in participants without DM, adjusted for included age, sex, stroke, WBC, ALB, TBIL, Scr, CO₂CP, Lac, CTnI, FIB, CRP, ABX<1 h, 24-h fluid balance, SOFA score, infection site, MAP, CHF, COPD, VAD, MV, and CRRT.

HR, hazard ratio; Ref, reference; CI, confidence.

In addition, due to the small sample size of Class 2, which may affect statistical power, and since multivariable Cox proportional hazards regression (Table 3, Model III) showed no statistically significant difference in mortality risk between Class 1 (persistently high PLT levels) and Class 2 (continuously increasing PLT count within 1 week) sepsis patients, we conducted a sensitivity analysis by combining these two trajectory classes as the reference group. In multivariable Cox proportional hazards regression, compared with the combined Classes 1 + 2, participants with gradually declining PLT trajectory (Class 3) and persistently low PLT trajectory (Class 4) had 1.68-fold (HR = 2.679, 95%CI: 1.027–6.990, *p* = 0.043) and 2.53-fold (HR = 3.528, 95%CI: 1.506–8.263, *p* = 0.004) increased mortality risk, respectively (Supplementary Table S1, Mode III). This is consistent with the results of multivariable Cox proportional hazards regression analysis (Table 4, Model III), indicating that sepsis patients with PLT levels that gradually decline from high baseline or remain persistently low during the first week may have significantly increased mortality risk.

Furthermore, sensitivity analyses further validated the robustness of the study findings. To assess the validity of multiple imputation data, we conducted a validation analysis based on the original data before imputation. Multivariate Cox proportional hazards regression showed that compared with Class 1 as the reference group, the HRs (95% CIs) for Class 2, Class 3, and Class 4 were 1.499 (0.204, 11.013), 4.860 (1.091, 21.657), and 5.031 (1.065, 23.771), respectively (Supplementary Table S2, Mode III). These results demonstrate consistency with the conclusions of the post-imputation analysis, confirming the credibility and validity of the multiple imputation analysis. Meanwhile, E-value analysis was performed to assess the potential impact of unmeasured confounding factors. The results showed that the E-values for each PLT change category (Class 2–4 vs. Class 1) were 2.23, 4.31, and 4.77, respectively, all of which exceeded the relative risks between PLT change categories and unmeasured confounding factors (1.72, 2.93, and 3.20 for Class 2–4 vs. Class 1, respectively), and were all smaller than the relative risks between unmeasured confounding factors and 28-day mortality (3.63, 9.57, and 10.99 for Class 2–4 vs. Class 1, respectively), indicating that unknown or unmeasured factors are unlikely to significantly influence the association between early dynamic PLT changes in sepsis and 28-day mortality. In conclusion, all sensitivity analyses confirmed that our study results demonstrated considerable robustness and reliability.

### Subgroup analysis

Supplementary Table S3 presented the relationship between the dynamic changes in PLT during the first week of admission and 28-day mortality among sepsis patients, stratified by potential risk factors. After adjusting for confounding variables, no significant interactions were observed between PLT dynamic changes and factors such as age, sex, shock, hypertension, CHF, CKD, and DM (P-interaction > 0.05). This indicates that these factors do not affect or modify the association between PLT dynamic changes and 28-day mortality (Supplementary Table S3).

## Discussion

This study employed dynamic latent class analysis to identify longitudinal PLT count trajectory patterns and validated their association with the prognosis of sepsis patients. The results revealed that sepsis patients whose PLT counts showed a marked downward trend from a relatively high level at admission during the first week had a higher 28-day mortality risk, while those whose PLT counts remained consistently low during the first week had the highest 28-day mortality risk. In addition, the GAMM model demonstrated that PLT counts in sepsis patients who did not survive for 28 days exhibited a progressive downward trend during the first week after admission.

Beyond their essential function in hemostasis, PLT also significantly contributes to inflammatory diseases (10). Research indicates that PLT acts as an immunogenic cell. Much like traditional innate immune cells, they are promptly recruited to sites of injury and inflammation, where they release immune mediators, express and shed immunologically active membrane receptors, interact with various immune cells, and recognize as well as eliminate pathogens (13). In the context of sepsis, the dysregulation of the complement, coagulation, and inflammatory systems, combined with PLT dysfunction, exacerbates tissue injury. The interplay between systemic inflammatory response syndrome and the compensatory anti-inflammatory response syndrome determines the clinical outcomes of sepsis (14). Additionally, thrombocytopenia is identified as an independent risk factor for increased mortality among patients with sepsis. Generally, 20–58% of sepsis patients experience thrombocytopenia, with severe thrombocytopenia occurring in 10% of cases (36). An analysis based on the publicly available database Medical Information Mart for Intensive Care IV (MIMIC-IV), involving 16,401 sepsis patients, found that PLT count categories within 24 h of admission (<50 × 10^9^/L, 50–100 × 10^9^/L, 100–150 × 10^9^/L, ≥150 × 10^9^/L) were associated with 28-day mortality. After multivariable adjustment, the HR (95% CI) for 28-day mortality were 2.31 (1.99–2.68), 1.34 (1.18–1.51), 1.00 (reference), and 1.17 (1.06–1.29), respectively (37). Another analysis based on public data from Netherlands showed that, after adjusting for potential confounding factors, each 10 × 10^9^/L increase in PLT count at admission was associated with a 1% reduction in the 30-day mortality risk for sepsis patients (odds ratio OR = 0.99, 95% CI: 0.98–0.99). Compared to participants with a normal PLT count range (150–400 × 10^9^/L), sepsis patients with thrombocytopenia (<150 × 10^9^/L) had a 20% higher 30-day mortality risk (OR = 1.20, 95% CI: 1.02–1.40) (38). A study conducted in the United States arrived at comparable findings, indicating that the mortality risk for sepsis patients with thrombocytopenia was 1.4 times greater than that of those with normal PLT counts, and their six-month survival probability was also diminished (39). Our study established a negative association between PLT count at admission and 28-day mortality in sepsis patients. Furthermore, it was observed that patients with a PLT count exceeding 200 × 10^9^/L experienced significantly lower mortality, aligning with previous studies.

However, it is worth noting that longitudinal data may provide more information about disease progression compared to absolute PLT counts at specific time points. Trajectory analysis can reveal hidden values in repeated measurements (40, 41). Additionally, studies have shown that PLT counts fluctuate over time, typically reaching their lowest point between the third and fifth days of hospitalization in sepsis patients. This suggests that in addition to baseline PLT counts, the trajectory of dynamic PLT changes over time may play a crucial role in determining patient prognosis. Therefore, we hypothesize that the early dynamic change in PLT counts has a certain prognostic value in sepsis. Unfortunately, there are currently few studies on the relationship between early PLT dynamics and sepsis prognosis, with only two studies based on public data available. This study included patients with sepsis from the MIMIC-IV and the eICU Collaborative Research Database (eICU-CRD) (22). Using the K-means clustering method, the change in PLT counts during the first 4 days after ICU admission was categorized into three types: (i) increasing type, where PLT counts increased after ICU admission; (ii) stable type, where PLT counts remained persistently low; and (iii) decreasing type, where PLT counts rapidly decreased after ICU admission. Multivariable logistic regression analysis showed that the 28-day mortality risk was significantly higher in patients with stable and decreasing PLT trajectories during the first 4 days after admission. In the MIMIC-IV cohort, the adjusted OR for stable and decreasing types compared to the increasing type were 1.451 (95% CI 1.201–1.753) and 2.424 (95% CI 1.962–2.995), respectively. In the eICU-CRD cohort, the adjusted OR for stable and decreasing types compared to the increasing type were 1.326 (95% CI 1.080–1.627) and 2.519 (95% CI 1.950–3.255), respectively (22). Another study using MIMIC-IV data involved 15,250 sepsis patients and identified four distinct trajectories of PLT count changes over 14 days: normal levels (phenotype 1), persistently low levels (phenotype 2), gradually increasing levels above the normal range (phenotype 3), and persistently high levels (phenotype 4). Multivariable regression analysis revealed that the 28-day mortality rate in phenotype 2 was significantly higher than in phenotype 1 (OR = 1.69, 95% CI: 1.47–1.94). Compared to phenotype 1, no significant differences in in-hospital mortality were observed for phenotypes 3 and 4. Although phenotype 4 showed an increased 28-day mortality rate (*p* < 0.05), the inverse probability weighting results adjusted by regression analysis found no statistically significant difference in 28-day mortality between phenotype 4 and phenotype 1 (*β* = −0.006, 95% CI: −0.04–0.002, *p* = 0.723) (23).

This study systematically analyses the early PLT count change trajectories in septic patients for the first time using DLCM and reveals a close association between these dynamic changes and the 28-day mortality of patients. The study found that patients with persistently high PLT levels (Class 1) had the lowest mortality within 28 days, while patients with initially high but gradually decreasing PLT levels (Class 3) had higher mortality rates. Patients with persistently low PLT levels (Class 4) had the highest 28-day mortality. Additionally, the study found that compared to Class 1 patients, sepsis patients with low baseline PLT levels that gradually increased (Class 2) had an HR of 1.687 (0.380, 7.494). The confidence interval shows no statistically significant difference between them, but there is an upward effect trend. The large confidence interval indicates considerable uncertainty in the true effect size, mainly due to the small sample size and limited events, resulting in insufficient statistical power and imprecise effect estimation. Future prospective studies with larger sample sizes should be conducted to validate these preliminary observations. This result differs from previous studies for several reasons: First, regarding research methodology, prior studies primarily employed K-means clustering analysis to classify the PLT changes of septic patients during the first 4 days of hospitalization. This static data-based clustering method requires high data integrity; if data for any given time point is missing, it cannot effectively capture the dynamic characteristics of PLT changes, potentially leading to the exclusion of some patients with missing repeated measurements, which may introduce bias. In contrast, this study utilized DLCM to classify the PLT change trajectories over a duration of 7 days, allowing for the capture of dynamic changes in PLT over time and latent class characteristics, as well as the ability to handle irregular time point data, thus providing more comprehensive clinical information. Second, regarding data sources, previous studies were mainly based on MIMIC and eICU-CRD databases, with data primarily from ICU in Western countries, while this study is based on the Chinese population, having regional and population specificity. Cultural and regional differences include different treatment philosophies (such as conservative fluid management), differences in family involvement in decision-making processes, different resource allocation patterns, baseline PLT levels, and drug response variations. These factors may influence PLT dynamic change patterns by affecting treatment intensity, drug selection, and intervention timing, and future studies are needed in different cultural backgrounds and regions. Third, this study exhibits differences in baseline characteristics, such as sex ratio and racial distribution, as well as in adjusted variables compared to previous studies. Additionally, this study conducted sensitivity analyses that particularly focused on participants without CKD and CHD, further confirming the stability of the relationship between PLT change trajectories and 28-day mortality. Considering that the small sample size of Class 2 might affect statistical power, we further combined Classes 1 and 2 as the reference group (class 1 + 2). The analysis found that compared to the class 1 + 2 participants, sepsis patients with PLT levels gradually declining from a higher baseline (Class 3) or persistently low during the first week (Class 4) had a higher mortality, validating the relative reliability of the results. Subgroup analyses indicated that factors such as sex, age, BMI, or alcohol consumption do not modify the relationship between early PLT change patterns in septic patients and 28-day mortality. Furthermore, this study applied the GAMM to compare the trajectories of PLT counts over time between the 28-day survivor and non-survivor groups. The results showed that the survivor group had a relatively high baseline PLT count, which exhibited an overall upward trend, while the non-survivor group had a relatively low baseline PLT count that gradually declined over the week following admission. This further demonstrates that septic patients with persistently low PLT levels and a significant downward trend in PLT counts over time face an extremely high risk of mortality. Therefore, this study not only enhances our understanding of dynamic PLT changes in septic patients but also provides new perspectives and tools for clinical practice, which may have significant potential importance for improving the diagnosis and treatment of septic patients. Our findings suggest that dynamic PLT change trajectories may be associated with patient risk stratification, potentially assisting clinicians in identifying patients at higher risk and informing personalized treatment strategies. However, further prospective studies are needed to validate these associations and determine their clinical utility.

In the four PLT trajectory patterns, declining or persistently low PLT counts were associated with increased 28-day mortality risk, while sepsis patients with increasing or persistently high PLT counts typically had better prognosis. Each specific PLT change pattern may reflect different pathophysiological processes. Class 1 (persistently high PLT levels) may reflect the integrity of host defense mechanisms, typically demonstrating lower inflammatory marker levels and better organ function, indicating low mortality risk (42). Class 2 (rising from low baseline levels) may be associated with infection control and bone marrow function recovery (43). The gradually declining PLT trajectory from high baseline (Class 3) may reflect PLT activation and subsequent consumption, related to increased tissue factor release and dysregulated inflammatory response, which is significantly associated with organ dysfunction and higher mortality (36, 44). Persistently low PLT counts (Class 4) reflect sustained PLT consumption and bone marrow suppression, which may be related to infection deterioration and the development of severe thrombotic complications (such as disseminated intravascular coagulation), indicating high mortality potential (45–47). Future studies need to simultaneously monitor PLT dynamic changes and key biomarkers, such as P-selectin and neutrophil extracellular traps (NETs), and D-dimer, to validate the associations between PLT change trajectories and corresponding pathophysiological mechanisms (48, 49).

The advantages of this study are as follows: First, there is a scarcity of research investigating the relationship between PLT longitudinal variation patterns and short-term mortality in sepsis patients. This is the first study to use a DLCM to classify early PLT dynamic changes in Chinese sepsis patients and explore their impact on short-term mortality. Second, we employed a GAMM to further explore the early PLT trajectory patterns of sepsis patients with different prognoses. Third, we utilized multiple imputation techniques to address the issue of missing data. This strategy is widely recognized for its ability to enhance statistical power and minimize bias caused by missing covariate data. Fourth, the study is based on a cohort of Chinese patients, providing an in-depth analysis of the association between PLT count trajectories and 28-day mortality risk. It offers clinicians a new perspective for personalized prognostic assessment and treatment, with significant clinical practice implications. Moreover, to ensure the robustness of our findings, we conducted comprehensive subgroup analyses and a series of sensitivity analyses. These included re-evaluating the association between PLT variation patterns and short-term mortality after excluding patients with CKD or CHD.

However, it is necessary to highlight several limitations of this study. First, this study is a single-center cohort study from China, with all participants being Chinese, which greatly limits the generalizability of our study results to other ethnic populations or regions. In the future, we plan to collaborate with researchers outside China to conduct multi-center, multi-ethnic prospective cohort studies to validate early PLT dynamic patterns in sepsis patients and their relationship with prognosis among populations with different genetic backgrounds. Secondly, although this study conducted repeated measurements, the sample size is still relatively limited, particularly with only 33 cases in Class 2 of PLT changes, which may affect statistical power and lead to inaccurate effect estimation. For this reason, we conducted sensitivity analysis to enhance the reliability of the results. As an exploratory study, this research has a certain reference value, and in the future, we plan to collaborate with other researchers to conduct prospective cohort studies to further validate these findings. Third, consistent with all observational studies, although we adjusted for known and available confounding factors, the possibility of unmeasured or unconsidered confounding variables remains. Nevertheless, we computed E-values to evaluate the potential influence of unmeasured confounders, which suggested that unmeasured confounding factors are unlikely to account for the observed results. Fourth, cultural and regional biases may alter PLT dynamic patterns by influencing treatment intensity, medication selection, and intervention timing, thereby limiting the generalizability of our findings to other healthcare systems and populations. Future validation studies are needed in different cultural backgrounds and healthcare systems to ensure the universality and clinical applicability of platelet trajectory classification and its relationship with sepsis patient outcomes. Fifth, despite the application of multiple imputation to address missing values in this study, high missing rates for certain variables, especially critical ones, could still lead to bias. Nevertheless, sensitivity analyses performed on the pre-imputation original data showed good concordance with post-imputation results, supporting the robustness of our findings. Future prospective studies are planned to collect more complete and comprehensive data. Finally, this observational study cannot establish a causal relationship between early dynamic changes in PLT and short-term mortality risk in sepsis patients; it can only confirm the association between the two.

## Conclusion

This study identified four distinct dynamic patterns of PLT count changes within the first week of admission among Chinese sepsis patients. Independent associations were found between PLT dynamic changes and 28-day mortality, with higher 28-day mortality observed in patients whose PLT counts were gradually declining from high baseline levels or were persistently low. Additionally, it was discovered that sepsis patients who died within 28 days had overall low PLT counts characterized by a downward trend during the initial 7 days. Dynamic monitoring of early changes in PLT, especially paying attention to patterns of decline from high to low or persistently low levels, can help clinicians more effectively identify high-risk patients and develop personalized treatment strategies, such as optimizing fluid management, adjusting antibiotic regimens, or considering other supportive therapies, thereby improving the prognosis of sepsis patients.

## Data Availability

The raw data supporting the conclusions of this article will be made available by the authors, without undue reservation.

## Glossary

COPD: chronic obstructive pulmonary disease
PLT: platelet
APACHE II: Acute Physiology and Chronic Health Evaluation II score
CRP: C-reactive protein
DLCM: dynamic latent class model
MAP: mean arterial pressure
CKD: chronic kidney disease
NEU: neutrophil count
BNP: brain natriuretic peptide
eICU-CRD: eICU Collaborative Research Database
FIB: fibrinogen
CRRT: Continuous Renal Replacement Therapy
BE: base excess
CHF: congestive heart failure
CO_2_CP: carbon dioxide combining power
cTnI: cardiac troponin I
TBIL: total bilirubin
NETs: neutrophil extracellular traps
LYM: lymphocyte count
VAD: vasoactive drugs
DD: D-dimer
MIMIC-IV: Medical Information Mart for Intensive Care IV
RDW: red blood cell distribution width
AST: aspartate aminotransferase
DM: diabetes mellitus
FIO2: fraction of inspired oxygen
MAR: missing at random
ICU: intensive care unit
24h_out: 24-h output
HGB: hemoglobin
CHD: coronary heart disease
GAMM: Generalized Additive Mixed Model
BUN: blood urea nitrogen
WBC: white blood cell count
MV: mechanical ventilation
CI: confidence interval
ALT: alanine aminotransferase
SOFA: Sequential Organ Failure Assessment score
Lac: lactate
PCT: procalcitonin
ABX < 1 h: use of antibiotics within 1 h
ALB: albumin
Scr: serum creatinine
HR: hazard ratio
24h_in: 24-h intake
Ref: reference
CI: confidence interval
HR: hazard ratio

## References

[1] Singer M Deutschman CS Seymour CW Shankar-Hari M Annane D Bauer M. The third international consensus definitions for sepsis and septic shock (sepsis-3). JAMA. (2016) 315:801–10. doi: 10.1001/jama.2016.0287 26903338 PMC4968574

[2] Cecconi M Evans L Levy M Rhodes A. Sepsis and septic shock. Lancet. (2018) 392:75–87. doi: 10.1016/S0140-6736(18)30696-2 29937192

[3] Rudd KE Johnson SC Agesa KM Shackelford KA Tsoi D Kievlan DR. Global, regional, and national sepsis incidence and mortality, 1990-2017: analysis for the global burden of disease study. Lancet. (2020) 395:200–11. doi: 10.1016/S0140-6736(19)32989-7 31954465 PMC6970225

[4] Markwart R Saito H Harder T Tomczyk S Cassini A Fleischmann-Struzek C. Epidemiology and burden of sepsis acquired in hospitals and intensive care units: a systematic review and meta-analysis. Intensive Care Med. (2020) 46:1536–51. doi: 10.1007/s00134-020-06106-2 32591853 PMC7381455

[5] Fleischmann-Struzek C Mellhammar L Rose N Cassini A Rudd KE Schlattmann P. Incidence and mortality of hospital- and icu-treated sepsis: results from an updated and expanded systematic review and meta-analysis. Intensive Care Med. (2020) 46:1552–62. doi: 10.1007/s00134-020-06151-x 32572531 PMC7381468

[6] Liu V Escobar GJ Greene JD Soule J Whippy A Angus DC. Hospital deaths in patients with sepsis from 2 independent cohorts. JAMA. (2014) 312:90–2. doi: 10.1001/jama.2014.5804 24838355

[7] Cavigioli F Viaroli F Daniele I Paroli M Guglielmetti L Esposito E. Neonatal early onset sepsis (eos) calculator plus universal serial physical examination (spe): a prospective two-step implementation of a neonatal eos prevention protocol for reduction of sepsis workup and antibiotic treatment. Antibiotics (Basel). (2022) 11:1089. doi: 10.3390/antibiotics11081089 36009958 PMC9405114

[8] Iwashyna TJ Ely EW Smith DM Langa KM. Long-term cognitive impairment and functional disability among survivors of severe sepsis. JAMA. (2010) 304:1787–94. doi: 10.1001/jama.2010.1553 20978258 PMC3345288

[9] McNamara JF Harris P Chatfield MD Paterson DL. Long term sepsis readmission, mortality and cause of death following gram negative bloodstream infection: a propensity matched observational linkage study. Int J Infect Dis. (2022) 114:34–44. doi: 10.1016/j.ijid.2021.10.047 34718157

[10] Mackman N Tilley RE Key NS. Role of the extrinsic pathway of blood coagulation in hemostasis and thrombosis. Arterioscler Thromb Vasc Biol. (2007) 27:1687–93. doi: 10.1161/ATVBAHA.107.141911 17556654

[11] Shen L Tao C Zhu K Cai L Yang S Jin J. Key platelet genes play important roles in predicting the prognosis of sepsis. Sci Rep. (2024) 14:23530. doi: 10.1038/s41598-024-74052-w 39384856 PMC11464784

[12] Clark SR Ma AC Tavener SA McDonald B Goodarzi Z Kelly MM. Platelet tlr4 activates neutrophil extracellular traps to ensnare bacteria in septic blood. Nat Med. (2007) 13:463–9. doi: 10.1038/nm1565 17384648

[13] Das UN. Infection, inflammation, and immunity in sepsis. Biomol Ther. (2023) 13:1332. doi: 10.3390/biom13091332 PMC10526286 37759732

[14] Wang Y Ouyang Y Liu B Ma X Ding R. Platelet activation and antiplatelet therapy in sepsis: a narrative review. Thromb Res. (2018) 166:28–36. doi: 10.1016/j.thromres.2018.04.007 29655000

[15] Larkin CM Santos-Martinez MJ Ryan T Radomski MW. Sepsis-associated thrombocytopenia. Thromb Res. (2016) 141:11–6. doi: 10.1016/j.thromres.2016.02.022 26953822

[16] Kerris E Hoptay C Calderon T Freishtat RJ. Platelets and platelet extracellular vesicles in hemostasis and sepsis. J Investig Med. (2020) 68:813–20. doi: 10.1136/jim-2019-001195 31843956

[17] Claushuis TA van Vught LA Scicluna BP Wiewel MA Klein KP Hoogendijk AJ. Thrombocytopenia is associated with a dysregulated host response in critically ill sepsis patients. Blood. (2016) 127:3062–72. doi: 10.1182/blood-2015-11-680744 26956172

[18] Vanderschueren S De Weerdt A Malbrain M Vankersschaever D Frans E Wilmer A. Thrombocytopenia and prognosis in intensive care. Crit Care Med. (2000) 28:1871–6. doi: 10.1097/00003246-200006000-00031 10890635

[19] Thiery-Antier N Binquet C Vinault S Meziani F Boisramé-Helms J Quenot JP. Is thrombocytopenia an early prognostic marker in septic shock? Crit Care Med. (2016) 44:764–72. doi: 10.1097/CCM.0000000000001520 26670473

[20] Brun-Buisson C Meshaka P Pinton P Vallet B. Episepsis: a reappraisal of the epidemiology and outcome of severe sepsis in french intensive care units. Intensive Care Med. (2004) 30:580–8. doi: 10.1007/s00134-003-2121-4 14997295

[21] Venkata C Kashyap R Farmer JC Afessa B. Thrombocytopenia in adult patients with sepsis: incidence, risk factors, and its association with clinical outcome. J Intensive Care. (2013) 1:9. doi: 10.1186/2052-0492-1-9 25810916 PMC4373028

[22] Wang K Lu D Wang F. Subphenotypes of platelet count trajectories in sepsis from multi-center icu data. Sci Rep. (2024) 14:20187. doi: 10.1038/s41598-024-71186-9 39215039 PMC11364765

[23] Wang Y Wu J Shao T Su D Ma X Yu Z. Prognostic implications of changes in platelet trajectories in patients with sepsis: a retrospective analysis using the medical information mart for intensive care-iv database. Shock. (2024) 63:371–8. doi: 10.1097/SHK.0000000000002493 39450919

[24] Asparouhov T Hamaker EL Muthén B. Dynamic latent class analysis. Struct Equ Model Multidiscip J. (2016):1–13. doi: 10.1080/10705511.2016.1253479

[25] Asparouhov T Hamaker EL Muthén B. Dynamic structural equation models. Struct Equ Model Multidiscip J. (2018) 25:359–88. doi: 10.1080/10705511.2017.1406803

[26] Reitz KM Kennedy J Li SR Handzel R Tonetti DA Neal MD. Association between time to source control in sepsis and 90-day mortality. JAMA Surg. (2022) 157:817–26. doi: 10.1001/jamasurg.2022.2761 35830181 PMC9280613

[27] Hua Y Wang R Yang J Ou X. Platelet count predicts mortality in patients with sepsis: a retrospective observational study. Medicine (Baltimore). (2023) 102:e35335. doi: 10.1097/MD.0000000000035335 37746944 PMC10519494

[28] Kim YC Song JE Kim EJ Choi H Jeong WY Jung IY. A simple scoring system using the red blood cell distribution width, delta neutrophil index, and platelet count to predict mortality in patients with severe sepsis and septic shock. J Intensive Care Med. (2019) 34:133–9. doi: 10.1177/0885066618787448 30021478

[29] White IR Royston P Wood AM. Multiple imputation using chained equations: issues and guidance for practice. Stat Med. (2011) 30:377–99. doi: 10.1002/sim.4067 21225900

[30] Groenwold RH White IR Donders AR Carpenter JR Altman DG Moons KG. Missing covariate data in clinical research: when and when not to use the missing-indicator method for analysis. CMAJ. (2012) 184:1265–9. doi: 10.1503/cmaj.110977 22371511 PMC3414599

[31] Hastie T Tibshirani R. Exploring the nature of covariate effects in the proportional hazards model. Biometrics. (1990) 46:1005–16. doi: 10.2307/2532444 1964808

[32] Forrest WF Alicke B Mayba O Osinska M Jakubczak M Piatkowski P. Generalized additive mixed modeling of longitudinal tumor growth reduces bias and improves decision making in translational oncology. Cancer Res. (2020) 80:5089–97. doi: 10.1158/0008-5472.CAN-20-0342 32978171

[33] Lin S Lai D He W. Association between hyperglycemia and adverse clinical outcomes of sepsis patients with diabetes. Front Endocrinol (Lausanne). (2022) 13:1046736. doi: 10.3389/fendo.2022.1046736 36699043 PMC9868443

[34] Mansur A Mulwande E Steinau M Bergmann I Popov AF Ghadimi M. Chronic kidney disease is associated with a higher 90-day mortality than other chronic medical conditions in patients with sepsis. Sci Rep. (2015) 5:10539. doi: 10.1038/srep10539 25995131 PMC4650757

[35] VanderWeele TJ Ding P. Sensitivity analysis in observational research: introducing the e-value. Ann Intern Med. (2017) 167:268–74. doi: 10.7326/M16-2607 28693043

[36] Assinger A Schrottmaier WC Salzmann M Rayes J. Platelets in sepsis: an update on experimental models and clinical data. Front Immunol. (2019) 10:1687. doi: 10.3389/fimmu.2019.01687 31379873 PMC6650595

[37] Wang D Wang S Wu H Gao J Huang K Xu D. Association between platelet levels and 28-day mortality in patients with sepsis: a retrospective analysis of a large clinical database mimic-iv. Front Med (Lausanne). (2022) 9:833996. doi: 10.3389/fmed.2022.833996 35463034 PMC9021789

[38] Wang J Zhou P Li X Zhou L Deng Z. Association between platelet count and 30-day in-hospital mortality among intensive care unit patients with sepsis: a multicenter retrospective cohort study. Front Med (Lausanne). (2024) 11:1444481. doi: 10.3389/fmed.2024.1444481 39902030 PMC11788309

[39] Sharma B Sharma M Majumder M Steier W Sangal A Kalawar M. Thrombocytopenia in septic shock patients--a prospective observational study of incidence, risk factors and correlation with clinical outcome. Anaesth Intensive Care. (2007) 35:874–80. doi: 10.1177/0310057X0703500604 18084977

[40] Gerig G Fishbaugh J Sadeghi N. Longitudinal modeling of appearance and shape and its potential for clinical use. Med Image Anal. (2016) 33:114–21. doi: 10.1016/j.media.2016.06.014 27344938 PMC5381523

[41] Zhao L Murray S Mariani LH Ju W. Incorporating longitudinal biomarkers for dynamic risk prediction in the era of big data: a pseudo-observation approach. Stat Med. (2020) 39:3685–99. doi: 10.1002/sim.8687 32717100 PMC8011834

[42] Zhong Y Zhong L Zhou Y Liao Y Deng J. Dynamic neutrophil-to-lymphocyte-platelet ratio trajectories predict 30-day and 1-year mortality in sepsis: a retrospective cohort study based on mimic-iv 2.2. BMC Infect Dis. (2025) 25:594. doi: 10.1186/s12879-025-10987-3 40275138 PMC12023530

[43] Nagaraju N Varma A Taksande A Meshram RJ. Bone marrow changes in septic shock: a comprehensive review. Cureus. (2023) 15:e42517. doi: 10.7759/cureus.42517 37637609 PMC10457471

[44] Giustozzi M Ehrlinder H Bongiovanni D Borovac JA Guerreiro RA Gąsecka A. Coagulopathy and sepsis: pathophysiology, clinical manifestations and treatment. Blood Rev. (2021) 50:100864. doi: 10.1016/j.blre.2021.100864 34217531

[45] Delabranche X Boisramé-Helms J Asfar P Berger A Mootien Y Lavigne T. Microparticles are new biomarkers of septic shock-induced disseminated intravascular coagulopathy. Intensive Care Med. (2013) 39:1695–703. doi: 10.1007/s00134-013-2993-x 23793890

[46] Unar A Bertolino L Patauner F Gallo R Durante-Mangoni E. Pathophysiology of disseminated intravascular coagulation in sepsis: a clinically focused overview. Cells. (2023) 12:2120. doi: 10.3390/cells12172120 37681852 PMC10486945

[47] Yaguchi A Lobo FL Vincent JL Pradier O. Platelet function in sepsis. J Thromb Haemost. (2004) 2:2096–102. doi: 10.1111/j.1538-7836.2004.01009.x 15613012

[48] Etulain J Martinod K Wong SL Cifuni SM Schattner M Wagner DD. P-selectin promotes neutrophil extracellular trap formation in mice. Blood. (2015) 126:242–6. doi: 10.1182/blood-2015-01-624023 25979951 PMC4497964

[49] Zhang C Shang X Yuan Y Li Y. Platelet-related parameters as potential biomarkers for the prognosis of sepsis. Exp Ther Med. (2023) 25:133. doi: 10.3892/etm.2023.11832 36845958 PMC9947577

