# Supplementary material for: Early dynamic changes in platelet counts and 28-day mortality in sepsis patients: a retrospective cohort study using dynamic latent class model and generalized additive mixture model analysis
Source: Front Med (Lausanne). 2025 Jul 9;12:1596134. doi: 10.3389/fmed.2025.1596134 (PMC12283649; doi:10.3389/fmed.2025.1596134)
Supplement: Supplementary file 1 [file Table_1.DOCX]

**Early dynamic changes in platelet counts and 28-day mortality in sepsis patients: A retrospective cohort study using dynamic latent class model and generalized additive mixture model analysis.**

**Running title: Platelet and mortality in sepsis**

Yong Han^1,2#^, Jie Liu^1#^, Zhenhua Huang^1,2#^, Haofei Hu^2#^, Haiyan Yin^1^*

^1^Department of Intensive Care Unit, The First Affiliated Hospital of Jinan University, Guangzhou, China.

^2^Department of Emergency, Shenzhen Second People's Hospital, The First Affiliated Hospital of Shenzhen University, Shenzhen 518035, Guangdong Province, China.

^3^Department of Nephrology, Shenzhen Second People's Hospital, The First Affiliated Hospital of Shenzhen University, Shenzhen 518035, Guangdong Province, China.

Yong Han, Jie Liu, Zhenghuan Huang, and Haofei Hu have contributed equally to this work.

*Corresponding author

Haiyan Yin

Department of Intensive Care Unit, The First Affiliated Hospital of Jinan University,

No.613 W.Huangpu Avenue,

Guangzhou 510630

Guangdong Province,

China.

[Haiyanyin1867@126.com](mailto:Haiyanyin1867@126.com).

Table S1 Class 1 and Class 2 were combined as the reference group to analyze the relationship between early dynamic platelet changes and 28-day mortality in sepsis patients

| Exposure | Model I (HR, 95% CI) p | Model I (HR, 95% CI) p | Model I (HR, 95% CI) p |
| --- | --- | --- | --- |
| Classification of Dynamic Changes in PLT within 7 days NEW | |  |  |
| Class 1+2 | 1.0 | 1.0 | 1.0 |
| Class3 | 3.861 (1.578, 9.446) 0.003 | 3.541 (1.440, 8.707) 0.006 | 2.679 (1.027, 6.990) 0.044 |
| Class 4 | 6.111 (2.887, 12.938) <0.001 | 5.515 (2.568, 11.845) <0.001 | 3.528 (1.506, 8.263) 0.006 |

Model I did not adjust for any covariates.

Model II adjusted for age and sex.

Model III adjusted for age, sex, stroke, WBC, ALB, TBIL, Scr, CO₂CP, Lac, cTnI, FIB, CRP, MAP , ABX<1h , 24-hour fluid balance, SOFA score, Infection site, CKD, DM, CHF, COPD, VAD, MV, and CRRT.

HR, hazard ratio; Ref: reference; CI: confidence.

Table S2 The association between early dynamic platelet changes in sepsis and 28-day mortality based on the original data before imputation.

| Exposure | Model I (HR, 95% CI) p | Model II (HR, 95% CI) p | Model III (HR, 95% CI) p |
| --- | --- | --- | --- |
| Classification of Dynamic Changes in PLT within 7 days | |  |  |
| Class 1 | Ref | Ref | Ref |
| Class 2 | 2.170 (0.543, 8.676) 0.273 | 2.096 (0.422, 10.408) 0.365 | 1.499 (0.204, 11.013) 0.691 |
| Class 3 | 5.286 (1.704, 16.391) 0.004 | 6.529 (1.866, 22.848) 0.003 | 4.860 (1.091, 21.657) 0.038 |
| Class 4 | 8.367 (3.013, 23.230) <0.001 | 7.891 (2.434, 25.584)<0.001 | 5.031 (1.065, 23.771) 0.041 |

Model I did not adjust for any covariates.

Model II adjusted for age and sex.

Model III adjusted for age, sex, stroke, WBC, ALB, TBIL, Scr, CO₂CP, Lac, cTnI, FIB, CRP, MAP , ABX<1h , 24-hour fluid balance, SOFA score, Infection site, CKD, DM, CHF, COPD, VAD, MV, and CRRT.

HR, hazard ratio; Ref: reference; CI: confidence.

Table S3 Stratified association between platelet dynamic change patterns and 28-day mortality in sepsis by age, sex, shock, hypertension, CHF, CKD, and DM.

|  | Change in the PLT, HR (95% CI) | | | | P for interaction |
| --- | --- | --- | --- | --- | --- |
| Subgroup | Class 1 | Class 2 | Class 3 | Class 4 |  |
| Participants(n) | 70 | 33 | 44 | 119 |  |
| Sex |  |  |  |  | 0.2855 |
| Male | Ref | 1.223 (0.096, 15.628) 0.877 | 4.696 (0.921, 23.956) 0.063 | 6.175 (1.400, 27.244) 0.016 |  |
| Female | Ref | 6.292 (0.501, 79.090) 0.154 | 1.554 (0.090, 26.805) 0.762 | 2.666 (0.306, 23.239) 0.375 |  |
| Age |  |  |  |  | 0.2437 |
| <65 | Ref | 0.032 (0.000, 50.741) 0.360 | 12.748 (0.490, 331.740) 0.126 | 0.506 (0.023, 11.248) 0.667 |  |
| ≥65 | Ref | 2.677 (0.449, 15.977) 0.280 | 3.324 (0.796, 13.878) 0.099 | 4.533 (1.208, 17.010) 0.025 |  |
| Shock |  |  |  |  | 0.8182 |
| No | Ref | 1.303 (0.057, 29.661) 0.868 | 4.013 (0.199, 81.073) 0.365 | 4.308 (0.510, 36.370) 0.180 |  |
| Yes | Ref | 1.745 (0.258, 11.788) 0.568 | 2.516 (0.515, 12.289) 0.254 | 3.723 (0.815, 16.999) 0.090 |  |
| Hypertension |  |  |  |  | 0.9549 |
| No | Ref | 2.087 (0.236, 18.430) 0.508 | 3.265 (0.490, 21.780) 0.222 | 4.189 (0.753, 23.316) 0.102 |  |
| Yes | Ref | 4.389 (0.452, 42.576) 0.202 | 6.657 (0.833, 53.222) 0.074 | 11.679 (1.706, 79.935) 0.012 |  |
| CHF |  |  |  |  | 0.1507 |
| No | Ref | 16.589 (1.087, 253.211) 0.043 | 5.372 (0.300, 96.246) 0.254 | 14.667 (1.972, 109.096) 0.009 |  |
| Yes | Ref | 5.074 (0.855, 9.202) 0.0671 | 4.442 (0.073, 8.676) 0.374 | 10.771 (0.538, 14.298) 0.767 |  |
| DM |  |  |  |  | 0.8507 |
| No | Ref | 1.515 (0.612, 14.317) 0.5408 | 1.300 (0.263, 6.423) 0.0478 | 2.406 (1.524, 11.052) 0.026 |  |
| Yes | Ref | 1.672 (0.305, 9.170) 0.554 | 1.840 (1.096, 7.593) 0.046 | 3.362 (1.330, 9.159) 0.006 |  |
| CKD |  |  |  |  | 0.7721 |
| No | Ref | 2.666 (0.166, 42.788) 0.489 | 2.261 (0.246, 20.751) 0.4707 | 2.799 (0.429, 18.277) 0.282 |  |
| Yes | Ref | 1.536 (0.342, 6.890) 0.575 | 3.341 (1.054, 10.592) 0.040 | 5.022 (1.752, 14.396) 0.002 |  |

Note 1: Above model adjusted for age, sex, stroke, WBC, ALB, TBIL, Scr, CO₂CP, Lac, cTnI, FIB, CRP, MAP , ABX<1h , 24-hour fluid balance, SOFA score, Infection site, CKD, DM, CHF, COPD, VAD, MV, and CRRT.

Note 2: In each case, the model is not adjusted for the stratification variable.

HR, Hazard ratios; CI: confidence, Ref: reference.
